# Transcriptome analysis of Callery pear (*Pyrus calleryana*) reveals a comprehensive signalling network in response to *Alternaria alternata*

**DOI:** 10.1371/journal.pone.0184988

**Published:** 2017-09-21

**Authors:** Jialiang Kan, Tingli Liu, Na Ma, Hui Li, Xiaogang Li, Jinyan Wang, Baolong Zhang, Youhong Chang, Jing Lin

**Affiliations:** 1 Institute of Horticulture, Jiangsu Academy of Agricultural Sciences/ Jiangsu Key Laboratory for Horticultural Crop Genetic improvement, Nanjing, China; 2 Institute of Biotechnology, Jiangsu Academy of Agricultural Sciences, Nanjing, China; Kunming University of Science and Technology, CHINA

## Abstract

The pear is an important temperate fruit worldwide that is produced by a group of species in the genus *Pyrus*. Callery pear (*Pyrus calleryana* Decne) is characterized by high resistance to multiple diseases, good adaptability, and high ornamental value, and is therefore widely planted in pear orchards for edible fruit production or as stock. Plant pathogens are a major threat to pear yield. Black spot disease, caused by the filamentous fungus *Alternaria alternata*, is one of the most serious diseases in pear. Elucidation of resistant genes to black spot disease is extremely important for understanding the underlying mechanisms as well as for the development of resistant cultivars. In this study, high-throughput single-strand RNA-sequencing was used to compare the transcriptome profiles of Callery pear leaves before and after *A*. *alternata* incubation for 7 days. The analysis yielded 73.3 Gb of clean data that were mapped onto the reference genome of the Chinese pear, and differentially expressed gene(DEG)s were identified with |log2FC| ≥ 1. Functional annotation demonstrated that black spot disease promoted great changes in the overall metabolism, and enrichment analysis of gene ontology terms showed that most of them are closely linked to signalling network and photosynthesis. Specifically, the genes included mainly transcription factors and genes involved in calcium signalling and ethylene and jasmonate pathways. Eight members of the ethylene response factor transcription factor gene family Group IX, including ERF1, ERF7, and ERF105, were up-regulated to 2.03–3.37-fold compared with CK, suggesting their role in the defence response to pathogen infection. Additionally, multiple transcription factors involved in biotic stresses, such as NAC78, NAC2, MYB44, and bHLH28, were up-regulated. Furthermore, we identified 144 long non-coding (lnc)RNAs, providing new insight into the involvement of lncRNAs in the response to black spot disease. Our study provides valuable data on the molecular genetics and functional genomic mechanisms of resistance to black spot disease in Callery pear. A good understanding of the molecular response to this disease will allow the development of durable and environmentally friendly control strategies.

## Introduction

The pear is an important fruit grown in Asia, Europe, and all other temperate regions worldwide, produced by a group of species in the genus *Pyrus*. *Pyrus* belongs to the subfamily Spiraeoideae in the family Rosaceae, which contains multiple fruits and flowers like apple, strawberry, and rose. The pear is a fruit with a delicate, pleasant taste and smooth texture and thus has a high customer acceptance. Approximately 22 million tons of pear fruit are produced yearly worldwide, representing 4% of all fruit production (FAOSTAT, http://faostat.fao.org/). The Japanese pear (*Pyrus pyrifolia* Nakai), European pear (*P*. *communis* L.), and Chinese pear (*P*. *bretschneideri* Rehd. and *P*. *ussuriensis* Maxim.) are three major edible species commercially grown for fruit production. In China, other species such as *P*. *hopeiensis*, *P*. *sinkiangensis*, *P*. *armeniacifolia*, *P*. *pseudopashia*, *P*. *xerophila*, *P*. *betulifolia* Bunge, *P*. *phaeocarpa* Rehder, Callery pear (*P*. *calleryana* Decne), and *P*. *pashia* Buch are cultivated regionally on a small scale. These pears are also planted as stock for grafting to enhance the resistance and tolerance of scion pear to biotic and abiotic stresses.

Callery pear is characterized by high resistance to multiple diseases, high adaptability, and high ornamental value. As a species native to China, it is widely used as graft stock in south China. In the early 20th century, the Callery pear was introduced into the US as graft stock in an aim to promote resistance to fire blight disease in the European pear, which was used as scion. In the following decades, the Callery pear was also used as an ornamental plant. In total, 155 thousand Callery pear seedlings were sold in the US in 1998, worth 30 million dollars [1]. Notably, as the Callery pear is well adapted to the US climate, it is now becoming an invasive plant.

Plant pathogens are a major threat to economically important crops worldwide. Black spot disease (BSD), caused by the filamentous fungus *Alternaria alternata* Keissler (previously *Alternaria kikuchiana* Tanaka), is among the most serious diseases that decrease pear production. Pear BSD was first reported by Tanaka in 1933 [2] and was later identified in France [3]. Fungi in the genus *Alternaria* produce host-selective toxins, the structures of which have been determined in Japanese pear, strawberry, tangerine, apple, tomato, and rough lemon pathotypes [4,5]. The fungus mainly infects leaves, fruits, and new shoots, and usually causes pear necrosis and defoliation, shortens the productive period, and decreases the post-harvest storage period. In 1988, a mutant of Japanese pear resistant to BSD was selected from the susceptible ‘Nijisseiki’ by chronic gamma irradiation [6]. Susceptibility to BSD is controlled by the single dominant gene A, and susceptible cultivars are heterozygous. To date, the homozygous dominant (A/A) genotype has not been identified in Japanese pear.

Although the genomes and transcriptomes of the Chinese pear and the European pear are publicly available, genomic information of Callery pear is lacking. To date, only around 5200 protein sequences are searchable in the NCBI GenBank (Dec 1^st^, 2016). Xu et al. (2015) reported 78,695 unigenes in Callery pear identified by high-throughput RNA sequencing (RNA-seq). Comparison with the reference genome and gene models available on the pear genome website (http://peargenome.njau.edu.cn) identified 218167 SNPs, 23248 indels, and 18322 alternative splicing events. Although the resistance of Callery pear to BSD was identified decades ago, its molecular mechanism remains unclear. This study aimed to assemble mRNAs and lncRNAs with reference to a related pear genome in order to identify DEGs in response to *A*. *alternata*.

## Materials and methods

### Plant materials

The Callery Pear seeds were collected from the Pear Germplasm Resource Center, Institute of Horticulture, Jiangsu Academy of Agricultural Sciences. The original source of these seeds were the inbred offspring of collected wild race from Shengzhou City, Zhejiang Province, China (E120.58, N30.01) in 2008. Seeds were rinsed with water, coated with wet sand, and stored in a refrigerator at 4°C on November 4, 2015. On March 14, 2016, the seeds were germinated in 30-cm-diameter plastic pots, which contained a 1:1:1 mixture of peat moss, perlite, and vermiculite. The seedlings were grown in the greenhouse at 25°C with a 14-h light/10-h dark photoperiod under a photon flux of 300 μmol m^-2^ s^-1^. The seedlings were irrigated with ¼ Murashige and Skoog nutrient solution under routine management. Seedlings at the nine-leaf stage were used for experiments.

### Inoculation of *A*. *alternata*

Seedlings were inoculated with spores of a virulent strain of *A*. *alternata* provided by Dr Youzhou Liu (Institute of Plant Protection, Jiangsu Academy of Agricultural Sciences, Nanjing, China). The isolate was cultured in potato dextrose broth without shaking at 25°C for 10 days. Mycelial mats were washed with tap water and maintained at 25°C in the dark. The spores formed were collected, suspended in distilled water, and diluted to approximately 5 × 10^5^ spores/mL by using a haemocytometer. The spore suspension was sprayed with a glass atomizer onto two young leaves detached from each plant. The inoculated leaves were incubated in a moist chamber at 25°C for 48 h. For each seedling, 50 μL conidia were daubed on the leave surfaces, and control leaves of each plant were mock-inoculated with sterile water. After 7 days, 3 leaves from 3 seedlings were pooled. The leaves were weighed, rinsed in water to remove contaminants, quickly dried with paper towels, quickly frozen in liquid nitrogen, and stored at –70°C until RNA extraction.

### RNA library construction and sequencing

From two replicate samples refers to before and after incubation, total RNA was extracted using the Total RNA Kit (Tiangen, Beijing, China) and digested with DNase I (Takara, Japan) after quality testing on the Agilent 2100 BioAnalyzer. The RNA was purified with Dynabeads Oligo dT (Life, USA), and 100 ng of purified mRNA was used to construct RNA libraries using NEBNext UltraTM RNA Library Prep Kit for Illumina (NEB, USA) according to the manufacturer’s instructions. Qubit testing, 2% gel testing, and high-sensitivity DNA chip testing were used to guarantee library quality. A 10 ng RNA library was cluster generated in a cBot instrument using the TruSeq PE Cluster Kit (Illumina, USA) and sequenced on Illumina Hiseq^™^ 2500 instrument by Nanjing Huasequen Biotechnologies Co, Ltd., China. Raw data of each sample were used for further analysis.

### Transcriptome assembly

Trimmomatic (v0.32) was used to trim low-quality reads in raw data to obtain clean reads [7]. Reads with ambiguous N and adapter sequences (forward adapter: GATCGGAAGAGCACACGTCTGAACTCCAG, reverse adapter: GATCGGAAGAGCGTCGTGTAGGGAAAGAG) were trimmed, and low-quality bases with a Phred score lower than 20 on each end of the read were eliminated. Additionally, reads of fewer than 50 nt were eliminated. The genomic sequences and gene structure annotation of Chinese pear (*P*. *bretschneideri* Rehd.) were used as a reference for read mapping and transcriptome assembly. Specifically, clean reads were mapped to the pear genome using Bowtie software (v2.1.0) [8], and gene expression was determined as RPKM (Reads Per Kilo bases per Million reads [9] using SAMtools (v0.1.19) [10] in reference to annotated gene models with a GFF format file.

### Digital gene expression tag profiling

For each sample, the reads were mapped onto the pear gene models using Bowtie software (v2.1.0). Gene expression was compared pair-wise between all time points for each cultivar using the EBSeq software [11]. The log2 of fold change (log2FC), p-value and q-value were calculated for all genes, and only transcripts with |**log2FC| ≥ 1** were considered to be differentially expressed. It is noteworthy that for very low RPKM values (<0.01), we considered these values as 0.01 in DEG analysis for convenience.

### Annotation and classification of genes

Gene annotations were obtained by BLASTP searching against the NR, KOG, KEGG, and TrEMBL databases with an E-value cut-off of 10^−5^. The protein functional annotation of the hits with the highest sequence similarity was chosen to represent the potential function of the sequence.

### Real-time quantitative (q)PCR validation

Ten genes responsive to BSD identified by RNA-seq were chosen for experimental validation by qPCR: *NAC78* (Pbr040795), *NAC2* (Pbr027956), *MYB44* (Pbr015309 and Pbr022028), *ERF1* (Pbr023899 and Pbr012024), *ERF5* (Pbr023902), *ERF105* (Pbr012685), *bHLH28* (Pbr018411), and *TCP15* (Pbr039105). The primers for these genes were designed by Beacon Designer 7 software. qPCR assays were performed with three biological and three technical replicates as previously reported. Total RNA was isolated from inoculated and control leaves using a TaKaRa MiniBEST Plant RNA Extraction Kit (9769), according to the manufacturer’s instructions. The RNA was used to synthesize first-strand cDNA using M-MLV reverse transcriptase (TaKaRa, Japan). The SYBR Premix Ex Taq II reagent (Takara, Japan) with SYBR Green I as the fluorescent dye was used for qPCR on an ABI 7300 real-time PCR system (Applied Biosystems, Foster City, CA, USA). Each reaction contained 10 μL 2× SYBR Premix Ex Taq II Reagent, 1.0 μL cDNA sample, and 500 nM gene-specific primers in a final volume of 20 μL. RNA levels were expressed relative to that of the actin gene (forward: GGAATGGTCAAGGCTGGGTT; reverse: CAAAGCATCTGTGAGGTCACG) as 2^–ΔΔCt^, where Ct is the cycle threshold, according to a previous workflow [12,13].

## Results

### Morphological changes in response to *A*. *alternata* and confirmation of disease establishment

Upon inoculation with 5 × 10^5^ spores/mL, all pear seedlings showed typical symptoms of black spots on the leaves after 7 days of incubation. PCR on cDNA generated from RNA extracted from inoculated Caller pear leaves with primers to detect the pathogen yielded an approximately 400-bp fragment, which was consistent with the 398-bp fragment in previous reports [14]. In addition, qPCR results showed that the expression of *PcNAC1* increased 1.86-fold after incubation, confirming successful disease establishment (data not shown). Thus, a time period of 7 days after incubation was selected for characterization of gene expression to explore the possible molecular mechanism of the resistance response to BSD.

### Sequencing and transcriptome assembly

To obtain a global overview of the Callery pear response to BSD, we constructed 4 RNA libraries containing mRNAs and lncRNAs, two replicates of which were constructed before incubation and two replicates were constructed after incubation. RNA sequencing produced 512.83 million 150-bp paired-end reads in total. After adapter removal and filtering steps, we obtained 73.3 Gb of clean data with more than 95.59% paired-end reads having a Phred score higher than Q30. All RNA-seq files are available from the NCBI database (accession number PRJNA393405).

High-quality filtered reads were used for reference-guided transcriptome assembly. The genome of the Chinese White pear was selected as the reference because it is closely related to Callery pear. The ratio of reads mapped to the Chinese pear genome was moderate, with a mapping rate between 61.54% and 65.06% (Table 1). This mapping ratio was close to that of 64.58% in a previous report [15]. For each sample, the mapped reads were assembled to genes according to the updated reference gene models, and the expression values were calculated for each gene and each isoform. The Chinese pear genome sequencing project predicted 42812 protein-coding genes, of which, on average, gene models consisted of transcript lengths of 2776 bp, coding lengths of 1172 bp, and 4.7 exons per gene. In this study, we obtained expression of 42812 genes (S1 Table). The average length of the assembled genes was longer than pervious study that adopted *de novo* assembly method.

**Table 1:** Statistics of sequencing data across the 4 libraries generated from Callery pear.

Sample	CK1	CK2	BSD1	BSD2
Raw reads (million)	153.70	160.72	99.54	98.85
Clean reads (million)	152.70	160.32	99.43	98.17
Raw data (Gb)	23.1	24.1	14.9	14.8
Clean data (Gb)	21.9	23.0	14.3	14.1
Left read Q20	99.69%	99.81%	99.80%	99.11%
Left read Q30	98.89%	98.71%	98.41%	98.85%
Right read Q20	98.77%	98.82%	98.77%	98.82%
Right read Q30	95.68%	95.59%	95.74%	95.88%
Total mapped (million)	98.99	104.30	61.19	61.15
Total mapped Ratio	64.83%	65.06%	61.54%	62.29%

### Identification of lncRNAs in Callery pear

**Fig 1:**
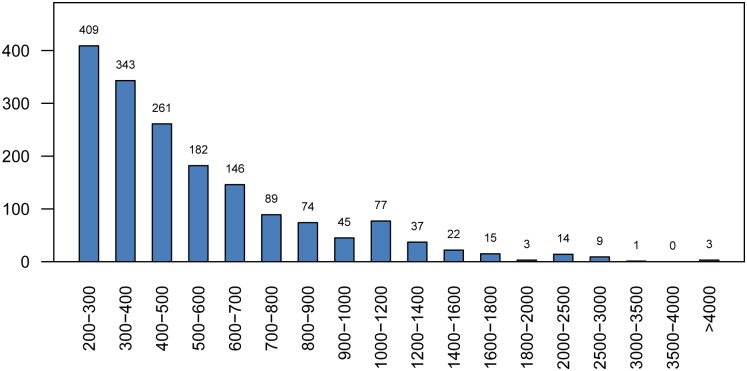
Length distribution of lncRNAs identified from the transcriptome of Callery pear.

The distinction of protein-coding from non-coding transcripts relies on the homologous alignment with well-studied proteins. Mapped reads that overlapped with coding gene models of Chinese pear were eliminated; the rest of the mapped reads that contained non-coding representative characteristics of other species were identified as lncRNA candidates. A large number of predicted and validated plant lncRNAs can be searched in public databases, such as the NONCODE [16] and PNRD [17] databases. In addition, the CPC (Coding Potential Calculator), CNCI (Coding-Non-Coding Index), and PLEK (predictor of long non-coding RNAs and messenger RNAs based on an improved k-mer scheme) pipelines are software programs designed to identify lncRNAs via computational approaches. CPC is a support vector machine-based classifier that can discriminate coding from noncoding transcripts with high accuracy [18]. CNCI was developed to effectively classify protein-coding or non-coding transcripts by profiling adjoining nucleotide triplets independent of known annotations [19]. PLEK is an effective predictor of lncRNAs and mRNAs using an improved k-mer scheme and a support vector machine algorithm [20]. Approximately 1730 novel transcripts were identified to be potential lncRNA transcripts by the intersection results of CPC, CNCI and PLEK software (S2 Table). The lncRNAs were shorter than coding RNAs, with an average length of 564 bp. The lengths of 409 lncRNAs fell into the range of 200 bp to 300 bp, 343 lncRNAs were 300 bp to 400 bp, and 261 lncRNAs were 400 bp to 500 bp. Among the latter, only 27 lncRNAs were longer than 2000 bp (Fig 1).

### DEGs in response to *A*. *alternata*

**Fig 2:**
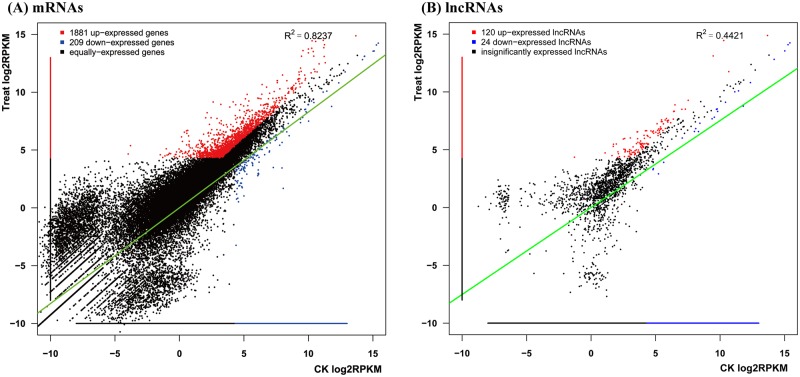
DEG statistical analysis of up-regulated and down-regulated genes in response to *A*. *alternata*.

To explore the molecular response to BSD, DEG analysis was performed. For each time point in two genotypes of Callery pear, three biological replicates were included. The *p*-value and q-value were calculated to measure the significance and reliability to reduce bias. In this study, DEGs were defined as those genes having a fold-change > 2 after eliminating bias on the basis of the three biological replicates (S3 Table). In total, 2090 DEGs were identified in response to *A*. *alternate* (Fig 2).

### Annotation of DEGs

The gene ontology (GO) consortium was set up to produce a dynamic, controlled vocabulary applicable to all eukaryotes even as the knowledge of gene and protein roles in cells is accumulating and changing [21]. In this study, GO enrichment analysis was carried out to clarify hierarchical relationships pertaining to molecular functions, cellular components, and biological processes of the 1191 DEGs in response to *A*. *alternata*.

**Fig 3:**
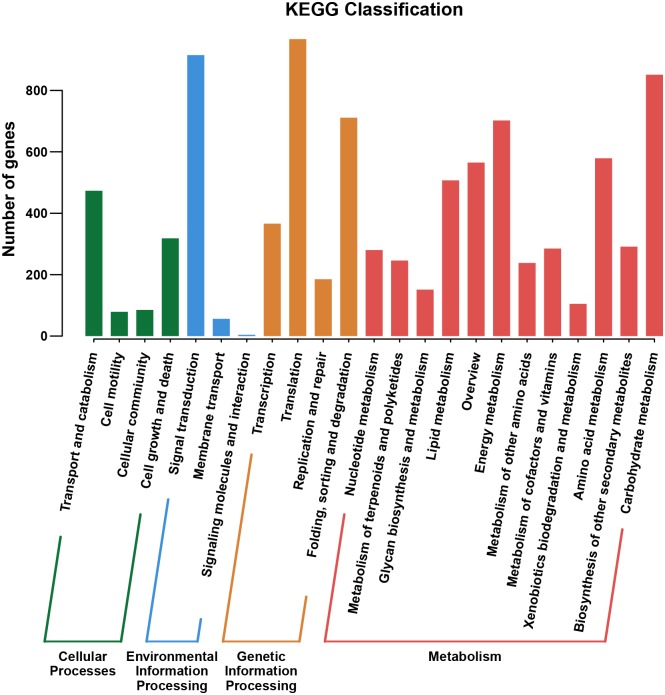
KEGG annotation of DEGs in response to *A*. *alternata*.

To better understand the biological functions of genes, we conducted KEGG pathway enrichment analysis. Based on sequence homology, DEGs were classified into 34 functional groups in 4 categories (Fig 3). In the report by Xu et al. (2015) on the response of Callery pear to salt stress, around 2100 genes were classified into the Signal Transduction functional group of the Environmental Information Processing category based on KEGG analysis. This study identified around 900 DEGs in this category, indicating the response of Callery pear to BSD shares some common features with that to abiotic stress. In the Metabolism category, genes involved in Carbohydrate Metabolism, Energy Metabolism, and Amino Acid Metabolism were the most abundant. In Genetic Information Processing category, over 900 genes were assigned to Translation and around 700 genes were assigned to Folding, Sorting and Degradation.

Furthermore, KOG terms were assigned to Callery pear genes based on the best BLAST hits from the KOG database. Out of the 2090 DEGs in total, 1066 DEGs (51%) could be assigned KOG terms. The three most represented categories were KOG1187 (serine/threonine protein kinase, 19 DEGs), KOG0540 (3-methylcrotonyl-CoA carboxylase, non-biotin containing subunit/acetyl-CoA carboxylase carboxyl transferase, subunit beta, 15 DEGs) and KOG1192 (UDP-glucuronosyl and UDP-glucosyl transferase, 14 DEGs).

### GO and KEGG enrichment analysis of DEGs

**Fig 4:**
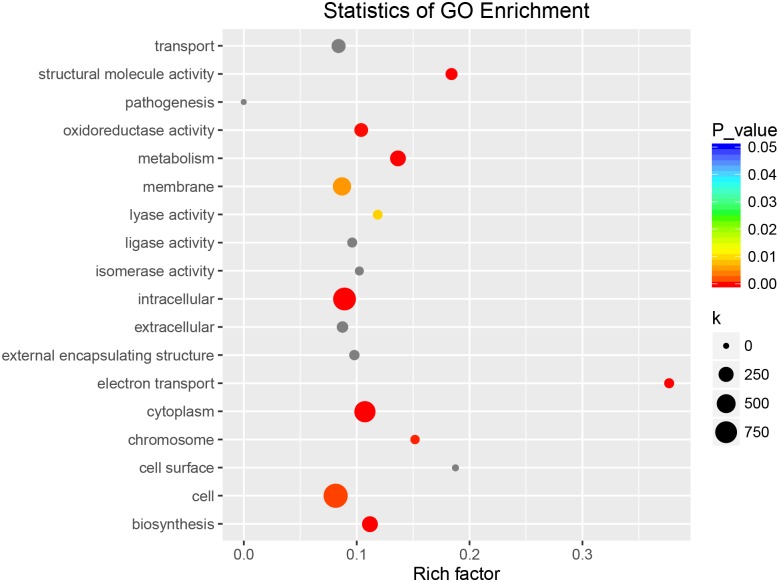
GO enrichment analysis of DEGs in response to *A*. *alternata*.

The above-mentioned GO annotation of DEGs was mainly focused on the accumulated number of DEGs in a distinct entry, ignoring the number of all genes in this species. Next, we performed KEGG enrichment analysis to unveil major pathways involved in the response to BSD based on the ratio of DEGs among all genes in each entry and on the *p*-value. In total, 1191 DEGs were assigned GO items and thus, were used in cluster frequency calculation. Correspondingly, 15348 were used as the denominator in the calculation as 15348 genes in Callery pear could be assigned GO items. The GO enrichment analysis showed that genes linked to electron transport, structural molecule activity, and cell surface were three most significant pathways in the response to *A*. *alternata* (Fig 4).

**Fig 5:**
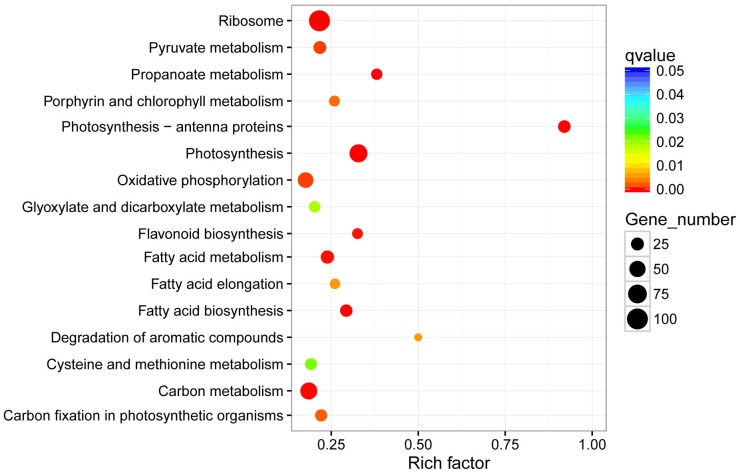
KEGG enrichment analysis of DEGs in response to *A*. *alternata*.

Similarly, 632 DEGs were annotated as functional enzymes involved in 23 pathways in the KEGG enrichment analysis (Fig 5). Interestingly, 23 out of 25 antenna protein (ko00196)-coding genes were differentially expressed, among 64 genes involved the photosynthesis pathway (ko00195), suggesting that photosynthesis is drastically deregulated in response to *A*. *alternata*. Carbon-related (ko01210, ko00190, and ko00710) and fatty acid metabolism-related (ko00061, ko01212, and ko00062) pathways were also differentially expressed in response to *A*. *alternata*. In addition, DEGs were also found in the flavonoid biosynthesis pathway.

### LncRNAs involved in the response of Callery pear to *A*. *alternata*

lncRNAs regulate target genes via *cis*- or *trans*-regulatory effects. Differentially expressed lncRNAs were selected for target prediction using the same criteria as DEGs. The genes that could be paired with lncRNAs transcribed within a 20-kbp window upstream or downstream of lncRNAs were considered as *cis* target genes [22]. RNAplex software was used to screen trans-acting target genes [23]. We identified 144 differentially expressed lncRNAs.

**Fig 6:**
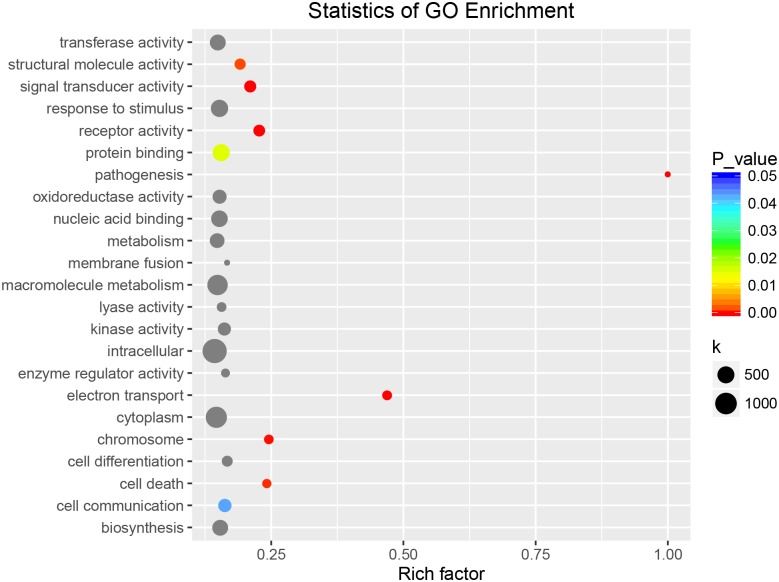
GO enrichment analysis of differentially expressed lncRNAs in response to *A*. *alternata*.

Interestingly, GO enrichment analysis results of targets of differentially expressed lncRNAs revealed their strong correlation with the DEGs. Ten functional groups, including Structural molecule activity, Pathogenesis, Oxidoreductase activity, Metabolism, Lyase activity, Intracellular, Electron transport, Cytoplasm, Chromosome, Biosynthesis, were also found in the GO enrichment analysis of Callery pear DEGs in response to *A*. *alternata* (Fig 6).

### qPCR validation

**Fig 7:**
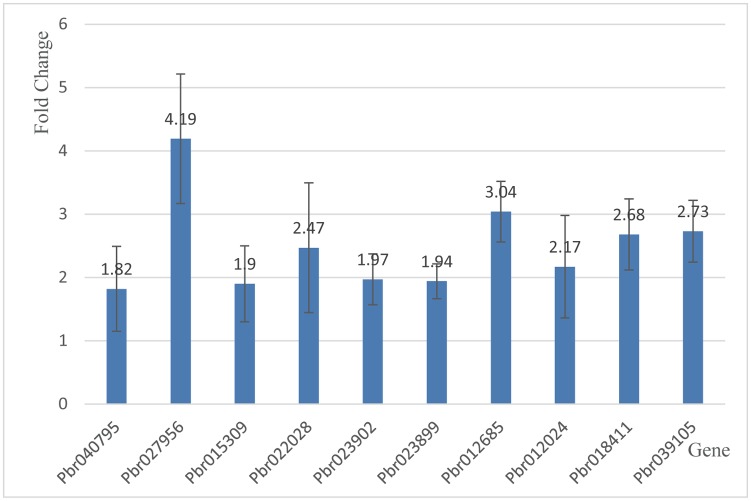
Relative expression of 10 selected DEGs in response to *A*. *alternata* assessed by qPCR. Error bars represent standard errors from three independent biological replicates.

To validate RNA-seq data, 9 genes were selected for analysis by qPCR. The correlation between the results was evaluated in R language using the RNA-seq fold-change values and relative expression levels quantified by qPCR. The Pearson correlation coefficient (r = 0.88) indicated that the gene expression levels obtained by qPCR and RNA-seq correlated, confirming the accuracy and reproducibility of the RNA-seq results (Table 2, Fig 7).

**Table 2:** DEGs in Callery pear in response to *A*. *alternata*.

Gene ID	Annotation	RNA-seq FC	qPCR FC	Sense Primer	Anti-sense Primer
Pbr040795	NAC domain-containing protein NAC78	2.30	1.82	TGACTTGACAGAGCACTA	CTTATTCTCAGTAGCATCCAA
Pbr027956	NAC domain-containing protein NAC2	4.58	4.19	TTGAGGCTTGATGATTGG	GTCCGATATTTCTGGGTATT
Pbr015309	MYB transcription factor MYB44	2.06	1.90	TGATAAGGAAGGAGGTGAG	TCAATTCTGCTAAACCCAAT
Pbr022028	MYB transcription factor MYB44	2.07	2.47	ATAAAGAACCACTGGAACTC	TTGAAATAGAGACCCGTAAC
Pbr023902	Ethylene-responsive transcription factor ERF5	2.11	1.97	CAATCTCGCTTCCTAACA	AGTGCTTCTTCTTCTCTTC
Pbr023899	Ethylene-responsive transcription factor ERF1A	2.15	1.94	GGAGTTCCAGCTTCAGTA	AACACCATAGAGCACCAT
Pbr012685	Ethylene-responsive transcription factor ERF105	2.42	3.04	GGAGGATGTTCAGGAGAT	TGAGGAGATAACGGAGAC
Pbr012024	Ethylene-responsive transcription factor ERF1, RAP23	2.52	2.17	GTAGTGACAAGTGATGAGAA	TCTGCCTTATTCCTCTGTA
Pbr018411	Basic helix-loop-helix transcription factor bHLH28	2.28	2.68	GACAACTTGGACAATCAGA	CCGAACCTACTATCTTCAC
Pbr039105	TCP transcription factor TCP15	3.34	2.73	ATCATCATCAGGCTGTTAC	GTGTTCGTGGTGAATACT

## Discussion

### Improvement of comparative transcriptome analysis of Callery pear

The current study had several innovations in experimental design and bioinformatics analysis as compared with the previous report on the transcriptome of Callery pear in response to salt stress by Xu et al. (2015). In the report by Xu et al., the mRNAs were first enriched and RNAs without poly(A) tails were eliminated by chemical methods before sequencing. In this study, only rRNAs and small RNAs were eliminated and mRNAs were not enriched, which means that a large amount of lncRNA fragments were retained in the RNA libraries. Moreover, Xu et al. assembled reads into genes by the *de novo* method. In contrast, as Callery pear is closely related to Chinese White pear, the genome of the latter was used for transcriptome assembly, resulting a more precise dataset of gene models.

### Signalling in Callery pear in response to *A*. *alternata*

Five major categories of pathogenicity genes in *A*. *alternata* comprise signalling genes, toxin production- and detoxification-related genes, genes encoding metabolic enzymes, genes involved in the generation of specific infection structures, and genes encoding cell wall-degrading enzymes [24]. The signalling response of plants to stress involves a comprehensive series of molecular expression changes to achieve effective regulation of the response. First, receptor proteins in the cell membrane perceive environmental stimulation, inducing second messengers such as Ca^2+^, ROS, and phosphoinositide. These second messengers regulate the intracellular content of Ca^2+^, which bind intracellular Ca^2+^ receptors to activate the phosphorylation cascade. Some genes subsequently induced participate in plant hormone synthesis pathways and influence the contents of abscisic acid (ABA), salicylic acid (SA), jasmonic acid (JA), and ethylene (ET), which in turn activate downstream signalling pathways. In response to salt stress, Callery pear activates Ca^2+^ signalling pathways mediated by calcineurin B-like proteins and their interacting kinases [15]. Annotation of DEGs revealed many genes encoding second messengers, plant hormone synthetic proteins, and transcription factors. CML27 (also termed CaM27, Pbr038859) was up-regulated 2.88-fold in response to *A*. *alternate* by RNA-seq technology; this gene is also involved in calcium signalling pathways in Callery pear plants in response to salt stress [15].

### Activation of ET biosynthesis in response to *A*. *alternata*

ET is an important plant hormone that is transported as its precursor, 1-aminoeyclopropane-l-carboxylic acid (ACC), throughout the plant. ACC synthase and ACC oxidase are two key enzymes that control ET biosynthesis in higher plants. ACC is produced from S-adenosylmethionine (SAM) by ACC synthase and is converted to ET by ACC oxidase (Arimura et al. 2005). SAM-dependent methyltransferases are crucial in metabolism, cell signalling, and epigenetic programming via methylation of small molecules [25]. The SAM-coding genes Pbr010088 and Pbr016813 were up-regulated 1.26- and 1.82-fold by RNA-seq technology, respectively.

### Activation of JA and SA pathways in response to *A*. *alternata*

JAs are plant signalling molecules that are systemically activated in response to various biotic and abiotic stresses [26]. Activation of the JA pathway increases the resistance of host plants to some pathogens, while activation of the SA pathway mediates resistance to other pathogens [27]. In this study, dozens of genes involved in JA metabolism were up- or down-regulated. Two genes (Pbr004541 and Pbr004568) encoding LOX2, which is involved in JA biosynthesis upon wounding or methyl jasmonate treatment in Arabidopsis [28], were increased over 1.5-fold in Callery pear in response to *A*. *alternata*. Lipid peroxidation is common to all biological systems and appears in the interaction of plants with pathogens, insects, and abiotic stresses, and at distinct stages of development. As ubiquitous proteins in plants, linoleate:oxygen oxidoreductases (LOXs) contribute to the oxygenation of linoleic acid leading to (13S)-hydroperoxy derivatives of poly-unsaturated fatty acids [29].

Pbr015509, which encodes PR protein B, was up-regulated 1.11-fold in this study. JA can induce major signalling components in the JA pathways, including F-box proteins and PR proteins. Thionins are small (5 kDa), usually basic, cysteine-rich peptides that are classified into the PR-13 family of PR proteins [30]. The expression of leaf thionins increases upon fungal infection in barley [31] and *A*. *thaliana* [32]. We identified only a few genes involved in SA signalling, indicating that SA signalling plays a limited role in the response of Callery pear to BSD.

### Transcription factors potentially involved in the resistance of Callery pear to BSD

In plants, transcription factors play a central role in synchronizing cell metabolism by self-regulation or by regulating the transcription of downstream target genes. Various transcription factors involved in biotic stress and plant development, including NAC, MYB, ERF, bHLH, and TCP, were found to be potentially involved in the response of Callery pear to *A*. *alternata*. We validated their expression by qPCR.

The ET-responsive transcription factor (ERF) family, which is part of the AP2/ERF superfamily, is a large gene family of transcription factors of about 60 to 70 amino acids in Arabidopsis [33]. Among over 100 members in Arabidopsis and rice, the ERF genes in group IX have often been associated with defence gene expression in response to pathogen infection [34]. Eight members of Group IX were up-regulated 1.03–2.37-fold in this study, including homologs of AtERF1 (Pbr023899, Pbr035787, Pbr012024, Pbr035775), AtERF5 (Pbr023902), AtERF9 (Pbr016222), and AtERF105 (Pbr012685), whereas no members were found to be down-regulated. ERF1 is associated with the molecular response and resistance to fungal infection. Overexpression of ERF1 in Arabidopsis enhanced resistance to two necrotrophic fungi, while it reduced resistance to *Pseudomonas syringae* [35]. ERF5 might act as a positive regulator of JA-mediated defence to *Botrytis cinerea*. Expression of ERF105 increases strongly in response to cold treatment as well as cold stress-related hormones in Arabidopsis [36]. Besides Group IX, Pbr024467, which is homologous to AtERF7 in Group VIII and plays an important role in the ABA response in plants [37], was up-regulated 1.44-fold in our study. Up-regulation of these genes was validated by qPCR, illustrating that ERF genes play an important role in the response of Callery pear to *A*. *alternata* and suggesting their involvement in the interaction networks between with ET, JA, and ABA signalling pathways.

MYB and MYB-like protein members were suggested to be involved in calcium signalling pathways in the salt-stress response in pear as indicated by high-throughput RNA-seq; however, their involvement was not verified [15]. In the current study, several proteins homologous to MYB4, MYB44, MYB308, MYB-like protein APL, MYB-like protein J, and MYB-like protein G were identified as being expressed in response to *A*. *alternata*. MYB4 (Pbr024492), which is orthologous to At1g22640, an SA- and JA-inducible transcription factor [38], was increased 16.61-fold. In rice, MYB4 represents a crucial node in the cross-talk of stress signalling cascades through the activation of multiple components, thus improving tolerance/resistance to multiple stress conditions such as drought, salt, UV, ozone, viruses, bacteria, and fungi [39]. In Arabidopsis, MYB44 belongs to the R2R3 MYB subgroup transcription factor family, which can be induced by ABA, methyl jasmonate, ET, and abiotic stresses such as dehydration, low temperature, and salinity [40]. qPCR results showed that two genes encoding MYB44 were up-regulated 0.90- and 1.47-fold as possible pathogen-responsive genes.

qPCR results confirmed that the *NAC2* gene, which is mainly activated by salt stress in Arabidopsis [41], was up-regulated over 3.19-fold in Callery pear by *A*. *alternata*. According to qPCR results, *NAC78* increased 0.82-fold; NAC78 has been implicated as a positive regulator of the expression of core 26S [42,43]. Moreover, it is involved in the regulation of gene expression related to flavonoid biosynthesis [44], which might explain why some genes involved in flavonoid biosynthesis were differentially expressed according to KEGG enrichment analysis.

Moreover, TEOSINTE BRANCHED1-CYCLOIDEA-PCF transcription factors (TCP), which play a vital role in the regulation of developmental processes and hormone responses, were differentially expressed in Callery pear upon BSD. TCP15 is a repressor of auxin biosynthesis, and regulates cytokinin and auxin responses during gynoecium development in Arabidopsis [45]. Additionally, TCP15 can modulate the expression of transcription factors involved in the induction of anthocyanin biosynthesis genes, thus inhibiting anthocyanin accumulation during exposure to high light intensity [46]. TCP15 (Pbr039105) was up-regulated 2.34-fold by RNA-seq and up-regulated 1.73-fold by qPCR. This suggested that TCP15 might act as positive regulator of the plant response to biotic stress.

### Genes linked to pathogenicity factors

A large number of genes in plants are linked to pathogenicity factors of fungal infection. For example, the plant *FRP1* gene is required for pathogenesis. The fungus *Fusarium oxysporum* completely loses pathogenicity in the absence of the *FRP1* gene in tomato, and fully regains virulence upon re-introduction of this gene. F-box proteins interact with S-phase kinase-associated protein 1 (Skp1) and facilitate targeting of proteins to the SCF—ubiquitination complex. SCF mediates ubiquitination in the infectious programme of *F*. *oxysporum* [24]. Five genes (Pbr005806, Pbr023291, Pbr034809, Pbr007369, and Pbr007370) encoding SCF ubiquitin ligase were up-regulated 1.22–2.19 fold after *A*. *alternata* invasion. In addition, 9 genes coding F-box proteins were identified, 8 of which were up-regulated.

## Conclusion

In conclusion, we performed a comprehensive analysis of the molecular response to *A*. *alternata*, the causative agent of BSD, using ssRNA-seq. In total, 1946 DEGs were identified. Additionally, 144 long non-coding RNAs were identified that might be involved in the response to *A*. *alternata*. This assembled and annotated transcriptome provides a valuable genomic resource for further investigation of the molecular mechanisms of resistance to BSD in Callery pear. A good understanding of molecular response to pathogens will allow the development of durable and environmentally friendly strategies for disease control.

## Supporting information

S1 Table The gene expression of Callery pear. (XLSX)

S2 Table The expression of lncRNAs in Callery pear. (XLSX)

S3 Table The expression and annotation of differentially expressed gene in response to fungus *Alternaria alternate*. (XLSX)
